# Effects of Black Cumin Seed Extract on Pancreatic Islet β-Cell Proliferation and Hypoglycemic Activity in Streptozotocin-Induced Diabetic Rats

**DOI:** 10.3390/antiox14020174

**Published:** 2025-01-31

**Authors:** Jongkyu Kim, Yoon-Seok Chun, Namkyu Yoon, Byungkwon Kim, Kiin Choi, Sae-Kwang Ku, Namju Lee

**Affiliations:** 1 AriBnC Co., Ltd., Yongin 16914, Republic of Korea; jkkim@aribnc.com (J.K.); ceochun@aribnc.com (Y.-S.C.); nkyoon@aribnc.com (N.Y.); kimbk8209@aribnc.com (B.K.); kichoi@aribnc.com (K.C.); 2 Department of Anatomy and Histology, College of Korean Medicine, Daegu Haany University, Gyeongsan 38610, Republic of Korea; 3 Intergrative Medicine Innovation Center, Wonkwang University, 460 Iksan-daero, Sindong, Iksan 54538, Republic of Korea

**Keywords:** thymoquinone (TQ), black cumin seed extract (BCS), antioxidant function, streptozotocin-induced diabetes, hyperglycemia management

## Abstract

Thymoquinone (TQ), a bioactive compound derived from black cumin seeds, is renowned for its potent anti-obesity and anti-diabetic properties. Due to the stability challenges of TQ, it has predominantly been utilized in oil formulations. This study aimed to enhance the stability of TQ and investigated the impact of consuming insoluble fiber from black cumin seeds on restoring antioxidant function compromised by diabetes and improving hyperglycemia management. We evaluated the restorative effects of a 35-day administration of black cumin seed extract (BCS) on antioxidant function impaired by streptozotocin (STZ)-induced diabetes, alongside structural and functional alterations in the pancreas, liver, and kidneys. The results demonstrated significant improvements in organ weight, particularly in pancreatic tissue. Moreover, BCS administration markedly suppressed the expression of key genes associated with pancreatic dysfunction and damage, including caspase-3, transforming growth factor-beta 1 (TGF-β1), and interleukin-1 beta (IL-1β). Through oral glucose tolerance tests (OGTTs), BCS was found to effectively regulate chronic hyperglycemia and exhibit potential for managing acute hyperglycemia. These findings suggest that BCS not only addresses both glycemic and non-glycemic complications of diabetes but also offers a safe, long-term solution. Consequently, BCS emerges as a promising therapeutic agent for hyperglycemia management, including in prediabetic stages.

## 1. Introduction

Diabetes is not only a leading cause of death worldwide but also a major health problem that significantly impacts the burden of disease. Moreover, the global prevalence of diabetes is continuously increasing. In South Korea, the prevalence of diabetes among individuals aged 19 and older is expected to rise by more than 20% by 2050 compared to the current rate [1]. Chronic hyperglycemia caused by diabetes not only leads to the overproduction of reactive oxygen species (ROS) but also induces oxidative stress [2], resulting in damage, dysfunction, and failure of various organs and tissues, as well as the development of microvascular (retinopathy, nephropathy, and neuropathy) and macrovascular (cardiovascular disorders) complications [3,4]. Therefore, since most diabetic complications can be managed through the control of hyperglycemia [2], synthetic drugs such as insulin secretagogues from the sulfonylurea class, dipeptidyl peptidase-4 inhibitors, metformin, thiazolidinediones, glucagon-like peptide-1 agonists, and sodium-glucose cotransporter-2 inhibitors are widely used. However, these drugs are associated with mild side effects like nausea and heartburn, as well as severe side effects such as hypoglycemia, angioedema, and gastrointestinal disturbances [5,6]. Consequently, there is growing scientific interest in the need for natural-product-based oral hypoglycemic agents that are not only safer but also more efficacious.

Black cumin (*Nigella sativa* L.) is an annual flowering plant of the Ranunculaceae family that has long been used as a medicinal herb, particularly in the Middle Eastern region and Western Asian countries, for the treatment of various conditions such as rheumatoid arthritis, asthma, inflammatory diseases, and diabetes mellitus [7,8]. Black cumin seed extract (BCS) is primarily utilized in the form of oil, with thymoquinone (TQ) as its main active compound, well-known for its anti-inflammatory and hypoglycemic pharmacological effects [9,10]. The TQ content in black cumin (BC) oil is reported to range between approximately 3.04 and 8.73 mg/g (0.30–0.87%) [11,12,13]. However, when black cumin seed fiber, generated during the oil extraction process, is recombined with the oil, the TQ content significantly increases to over 55 mg/g (5.5%) [14]. This enhancement is expected to improve the efficacy of the human equivalent dose in terms of its anti-inflammatory and hypoglycemic pharmacological effects. Additionally, the insoluble fiber in BC not only aids in cholesterol regulation [15] but also contributes to blood glucose control [16]. Therefore, when using BCS for blood glucose management, it is anticipated that both TQ and BC fiber play synergistic roles in achieving the desired therapeutic effects.

Using a high-fat-diet mouse model, we observed significant improvements in type 2 diabetes symptoms over a 12-week period following the administration of BCS containing insoluble fiber (TQ, 58.53 mg/g). These improvements included changes in blood insulin levels, HbA1c, blood glucose, hepatic glucose-regulating enzymes, and pancreas weight, suggesting that it may effectively control hyperglycemia symptoms caused by the loss of pancreatic islet β-cell function [14]. However, although several previous studies [8,17] have reported the anti-diabetic and hypoglycemic effects of BC oil using streptozotocin (STZ)-induced animal models, the underlying mechanisms responsible for its hyperglycemia-controlling effects remain unclear.

Streptozotocin (STZ) is widely used in diabetes research as it selectively destroys the insulin-producing β-cells in pancreatic islets, thereby inducing diabetes [18]. In this study, we investigated the anti-hyperglycemic effects of BCS using STZ-induced diabetic rats and explored the potential underlying mechanisms. Notably, our findings confirmed that the combined use of BC oil and insoluble fiber is effective in the treatment of diabetes. This study provides valuable insights into the therapeutic and regulatory potential of BCS for hyperglycemia management, highlighting its possible role as an alternative or complementary approach to conventional therapies.

## 2. Materials and Methods

### 2.1. Experimental Design

Seventy-seven healthy male SPF/VAF outbred Crl:CD Sprague Dawley rats (6 weeks old) weighing 170–210 g upon arrival were obtained from OrientBio (Seungnam, Republic of Korea). All animals were housed under standard laboratory conditions (temperature: 22 ± 2 °C; relative humidity: 50 ± 10%; 12 h light/dark cycle) in individually ventilated cages. Certified rodent diet and water were provided ad libitum. Animals were acclimatized for 8 days before the experiment to ensure baseline health.

Diabetes was induced using a single intraperitoneal injection of STZ at a dose of 60 mg/kg of body weight dissolved in freshly prepared 50 mM citrate buffer (pH 4.5). Injection volumes were calculated based on individual body weights. The development of diabetes was confirmed one week after STZ injection by measuring fasting blood glucose levels using a glucometer from tail vein blood. Rats with fasting glucose levels >280 mg/dL were considered diabetic and included in the study.

**Figure 1:**
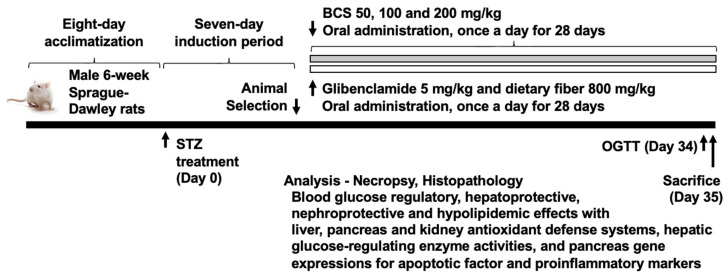
Experimental design of the study.

Diabetic rats were randomized into seven experimental groups (*n* = 10 per group): intact control (non-diabetic, untreated), STZ control (diabetic, untreated), glibenclamide treatment (5 mg/kg body weight, oral), dietary fiber treatment (800 mg/kg body weight, oral), BCS 50 mg/kg (low dose), BCS 100 mg/kg (medium dose), and BCS 200 mg/kg (high dose). Treatments were administered orally once daily via gavage for 28 consecutive days. The rats were weighed weekly, and gavage volumes were adjusted based on individual weights to ensure consistent dosing (Figure 1) (Table S1).

### 2.2. Test Materials

The BCS used in this study was supplied by AriBnC (Yongin, Korea) as a grayish-brown powder. Crushed BCS was mixed with 0.12% (*w/w*) rosmarinic acid, and the seed extract was prepared using a supercritical fluid extraction technique at 300 bar [14]. After preparing the seed extract, black cumin dietary fiber was added to the remaining free-flowing powder. The black cumin seed supercritical water extract (30–35%, *w/w*) was then mixed and homogenized with 50–52% (*w/w*) black cumin dietary fiber and 15–16% (*w/w*) magnesium carbonate, followed by lyophilization at −85 °C to produce the BCS used in this study. Glibenclamide (Sigma-Aldrich, St. Louis, MO, USA) and commercial dietary fiber (FIBER EAST™, containing 77% natural corn starch dietary fiber, Korea Medical Foods, Seoul, Republic of Korea) were used as reference controls. The dietary fiber was dissolved in distilled water at a concentration suitable for the 800 mg/kg dose.

### 2.3. High-Performance Liquid Chromatography Analyses

**Figure 2:**
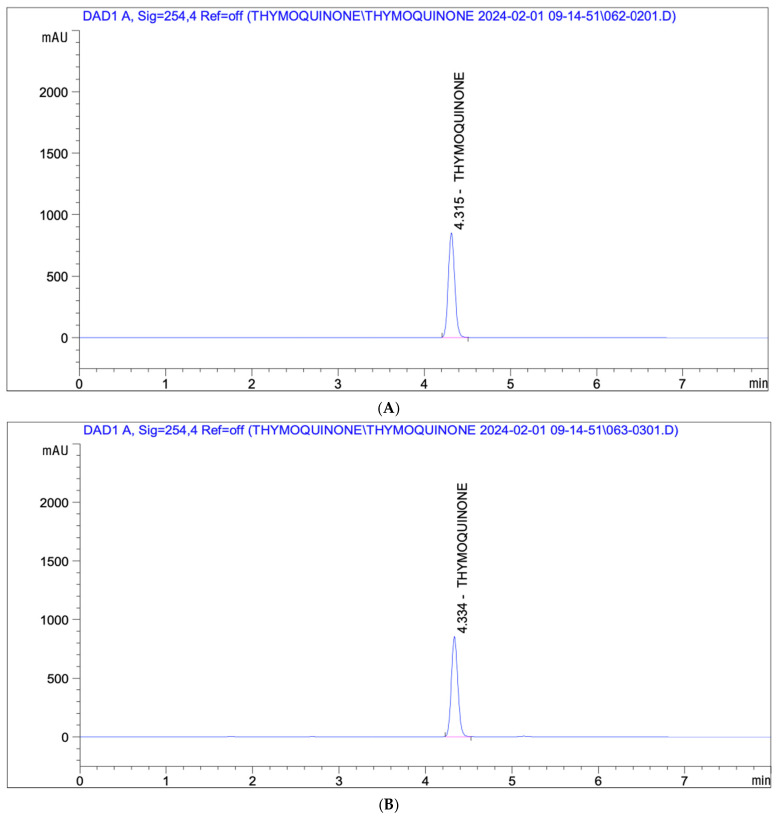
HPLC analysis of standard thymoquinone (**A**) and thymoquinone in the BCS (**B**).

The thymoquinone content of BCS was quantified using a UV–visible absorbance detector (Agilent Technologies, Inc., Santa Clara, CA, USA) coupled with a Capcell Pak C18 MG II column (4.6 mm × 250 mm, 5 μm, Osaka Soda Co., Ltd., Osaka, Japan) on an Agilent HPLC 1100 series (Agilent Technologies, Inc., Santa Clara, CA, USA). BCS and standard thymoquinone (Wuhan Chem Norm Biotech Co., Ltd., Wuhan, China) were dissolved in acetonitrile, and the solutions were filtered through a 0.45 μm membrane filter before injection. The column was maintained at 30 °C during the analysis, and thymoquinone was detected at 254 nm. The mobile phase consisted of distilled water and acetonitrile (18:82), and 10 μL of each sample was injected at a flow rate of 0.9 mL/min. The thymoquinone content was determined to be 58.04 mg/g (5.804%) (Figure 2).

### 2.4. Measurements

#### 2.4.1. Body Weight Measurement

Changes in body weight were assessed using an autonomic electronic balance (Model XB320M, Precisa Instrument, Zürich, Switzerland) from day −1, before STZ treatment, and then at 7-day intervals over a 35-day period. Additionally, body weight gains were calculated using Equation (1).

(1) $$\begin{array}{c} \mathrm{Body}\;\mathrm{weight}\;\mathrm{gains}\;\mathrm{during}\;7\;\mathrm{days}\;\mathrm{of}\;\mathrm{hyperglycemia}\;\mathrm{induced}\;\mathrm{periods}\\ \;\;\;=\mathrm{Body weight as initial test material oral administration}\\ \;\;\;-\mathrm{Body weight at the day of STZ treatment}\\ \mathrm{Body weight gains during 28 days of test article treatment periods}\\ \;\;\;=\mathrm{Body weight as sacrifice}-\mathrm{Body weight at initial test article treatment} \end{array}$$

#### 2.4.2. Organ Weight Measurement

At the time of sacrifice, 24 h after the final test article administration, the absolute weights of the liver, left kidney, and pancreas were assessed. Relative weight (percentage of body weight) was then calculated, taking into account changes in body weight, using Equation (2).

(2) $$Relative\;organ\;weight\;\left(\%\right)=\frac{absolute\;organ\;weight}{body\;weight\;at\;sacrifice\;}\times 100$$

#### 2.4.3. Measurement of Blood Glucose Level

Blood glucose levels were measured using plasma obtained from the right orbital plexus and analyzed with an automated blood analyzer (Dri-Chem NX500i; Fuji Medical System Co., Ltd., Tokyo, Japan).

#### 2.4.4. Serum Insulin and HbA1c

Blood HbA1c levels were analyzed using an automated HbA1c measuring system (Easya1c, Infopia, Anyang, Republic of Korea), while serum insulin levels were assessed using a rat insulin ELISA kit (Cat. No. MBS045315, Mybiosource, San Diego, CA, USA) [14].

#### 2.4.5. Serum Biochemistry

Blood samples were collected from the retro-orbital sinus under light anesthesia (Becton Dickinson, Franklin Lakes, NJ, USA) at the end of the study. Serum was separated by centrifugation at 3000× *g* for 10 min at 4 °C. Biochemical analyses were performed to measure insulin, AST, ALT, ALP, LDH, GGT, BUN, creatinine, total cholesterol, triglycerides, LDL, and HDL using a clinical chemistry analyzer with commercially available kits.

#### 2.4.6. Oral Glucose Tolerance Test (OGTT)

The oral glucose tolerance test (OGTT) was performed after the final treatment dose. Following an overnight fast, rats were orally administered D-glucose (3 g/kg body weight) (Cat. No. G8270; Sigma-Aldrich, St. Louis, MO, USA), which was dissolved in sterile distilled water and administered at a dose of 5 mL/kg of body weight. Blood samples were collected as 0.1 mL of whole blood from the right orbital plexus and then centrifuged at 4 °C for 10 min at 3000 rpm using a cryocentrifuge (Labocene 1236 MGR, Gyrozen, Daejeon, Republic of Korea). The plasma was separated and stored at −150 °C in an ultradeep freezer until analysis. Blood glucose levels were measured at 0, 30, 60, 90, and 120 min post-glucose administration using a glucometer. The percentage of variation in glycemia was calculated using Equation (3) [19]. The area under the curve (AUC) was calculated using the APL pharmacokinetic modeling program V.1.03.50 for Windows (APL Analyst, MN, USA).

(3) $$Blood\;glucose\;levels\left(\%\right)=\frac{blood\;glucose\;levels\;of\;samples}{baseline}\;\times \;100$$

#### 2.4.7. Pancreas Capase-3, TGF-β1, and IL-1β Gene Expression 

The mRNA expression of the apoptotic factor caspase-3, fibrosis marker TGF-β1, and proinflammatory marker IL-1β in the pancreas was measured using real-time (RT)-PCR (Bio-Rad, Hercules, CA, USA), as described in previous studies [20]. Expression patterns were compared to the mRNA expression of glyceraldehyde-3-phosphate dehydrogenase (GAPDH), which is commonly used as a standard. Gene expression was calculated using the comparative CT method described by Schmittgen and Livak [21]. The oligonucleotides used in this study are listed in Table S2.

#### 2.4.8. Histopathology and Immunohistochemistry

Pancreas, liver, and kidney tissues were excised, fixed in 10% neutral-buffered formalin, and embedded in paraffin. Sections (4–5 µm thick) were stained with hematoxylin and eosin (H&E) for histopathological evaluation. For immunohistochemical analysis, pancreatic sections were stained for insulin- and glucagon-producing cells using primary antibodies and visualized with an appropriate secondary antibody detection system.

#### 2.4.9. Oxidative Stress Markers

Lipid peroxidation was assessed by measuring malondialdehyde (MDA) content using the thiobarbituric acid reactive substance (TBARS) assay. Reduced glutathione (GSH) levels and activities of catalase (CAT) and superoxide dismutase (SOD) were determined in tissue homogenates using spectrophotometric methods.

#### 2.4.10. Hepatic Glucose-Regulating Enzymes

Activities of glucokinase (GK), glucose-6-phosphatase (G6pase), and phosphoenolpyruvate carboxykinase (PEPCK) were assessed in liver homogenates using enzymatic assays. Protein concentrations were determined using the Bradford method to normalize enzyme activities.

### 2.5. Statistical Analysis

Data were analyzed using SPSS software (Version 14.0K, IBM SPSS Inc., Armonk, NY, USA). Normality was assessed with the Shapiro–Wilk test, and homogeneity of variances was evaluated using Levene’s test. Inter-group comparisons were performed using one-way analysis of variance (ANOVA), followed by Tukey’s post hoc test for normally distributed data. When homogeneity of variances was not assumed, Dunnett’s T3 test was applied. All results are presented as the mean ± standard deviation (SD), and statistical significance was set at *p* < 0.05.

## 3. Results

### 3.1. Effects of BCS on Body Weight and Weight Gains in STZ-Induced Diabetic Rats

In the STZ-induced diabetic control group, a significant reduction in body weight was observed over the 28-day BCS administration period compared to the intact vehicle control group (*p* < 0.01). Glibenclamide (5 mg/kg) treatment resulted in a significant weight gain compared to the STZ-induced diabetic control group starting from day 21 (*p* < 0.01). Similarly, BCS at doses of 50, 100, and 200 mg/kg showed significant weight gain compared to the STZ-induced diabetic control group, beginning on days 21, 21, and 15, respectively (*p* < 0.01) (Figure S1). Additionally, over the 28-day administration period, the percentage of weight gain compared to the STZ-induced diabetic control group was as follows: glibenclamide (5 mg/kg), 165.75%; dietary fibers (800 mg/kg), 124.74%; BCS (50 mg/kg), 169.58%; BCS (100 mg/kg), 169.58%; and BCS (200 mg/kg), 224.37%.

### 3.2. Anti-Diabetic Hypoglycemic Effects

#### 3.2.1. Effects on Pancreatic Weight in STZ-Induced Diabetic Rats

In the STZ-induced diabetic control group, the absolute weight of the pancreas was significantly reduced compared to the intact vehicle control group (*p* < 0.01). However, treatments with glibenclamide (5 mg/kg), dietary fibers (800 mg/kg), and BCS (50, 100, and 200 mg/kg) significantly increased the absolute pancreatic weight compared to the STZ-induced diabetic control group (*p* < 0.05). For the relative pancreatic weight, only BCS at a dose of 200 mg/kg showed a significant increase compared to the STZ-induced diabetic control group (*p* < 0.05), while glibenclamide (5 mg/kg) and dietary fibers (800 mg/kg) did not show significant differences (Table 1).

#### 3.2.2. Reduction in Hyperglycemia Following BCS Administration

**Figure 3:**
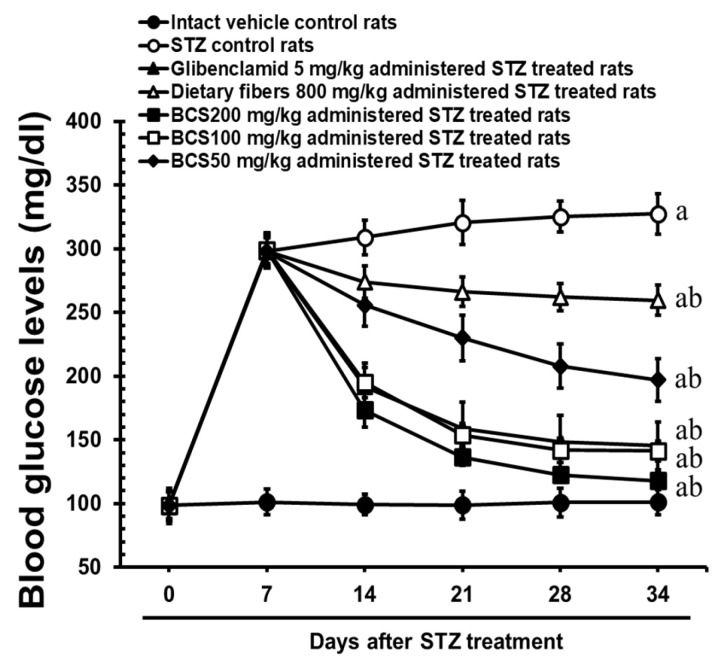
Dose-dependent effects of BCS on blood glucose levels (values are expressed as the mean ± SD of 10 rats). ^a^ *p* < 0.01 as compared with intact vehicle control by THSD test; ^b^ *p* < 0.01 as compared with STZ control by THSD test.

**Figure 4:**
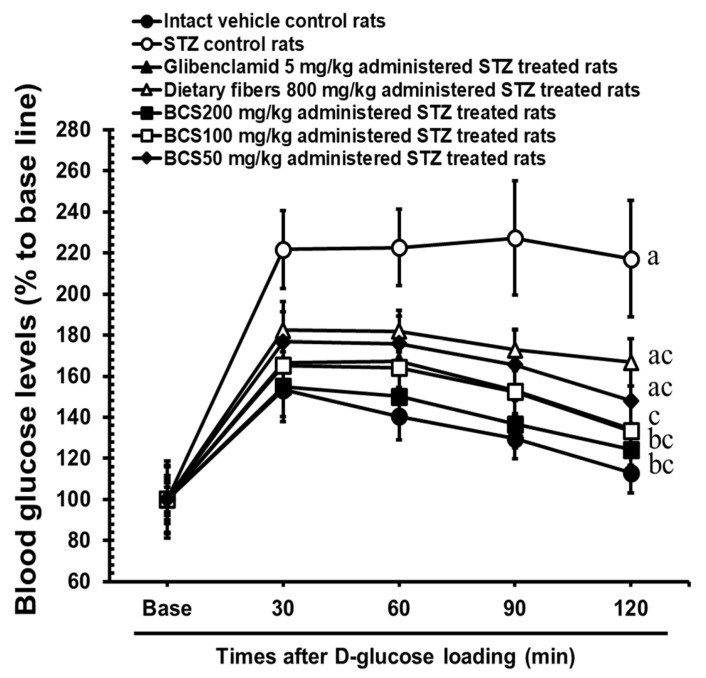
Oral glucose tolerance test (values are expressed as the mean ± SD of 10 rats). ^a^ *p* < 0.01 and ^b^ *p* < 0.05 as compared with intact vehicle control by DT3 test; ^c^ *p* < 0.01 as compared with STZ control by DT3 test.

The BCS administration significantly reduced fasting blood glucose levels in a dose-dependent manner across all BCS-treated groups. At the medium dose of 100 mg/kg, BCS demonstrated glucose-lowering effects that were comparable to those of glibenclamide (5 mg/kg), a well-established insulin secretagogue (Figure 3 and Figure 4). The 100 mg/kg BCS group showed a reduction in fasting blood glucose levels from 299.10 ± 10.92 mg/dL at 7 days after STZ treatment to 141.90 ± 9.56 mg/dL after 28 days of treatment, which was statistically significant when compared to the STZ-induced diabetic control group (*p* < 0.01). In contrast, the STZ group showed a progressive increase in blood glucose levels throughout the treatment period.

#### 3.2.3. Oral Glucose Tolerance Test (OGTT) in STZ-Induced Diabetic Rats Treated with BCS

**Figure 5:**
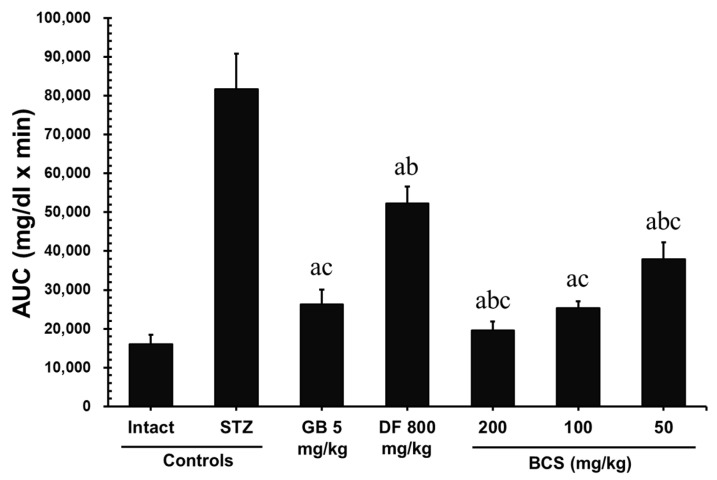
Effects of BCS treatment on the area under the curve (AUC) of the oral glucose tolerance test (OGTT) in STZ-induced diabetic rats that received 28 days of supplementation. ^a^ *p* < 0.05 as compared to the STZ control group; ^b^ *p* < 0.05 as compared to glibenclamide (5 mg/kg); ^c^ *p* < 0.05 as compared to dietary fiber (800 mg/kg).

After 28 days of BCS administration, an oral gavage of D-glucose (3 g/kg) was conducted, and blood glucose levels were measured at 30, 60, 90, and 120 min. Compared to the STZ-induced diabetic control group, significant reductions in blood glucose levels were observed in the BCS-treated groups at doses of 50, 100, and 200 mg/kg (*p* < 0.01). The area under the curve (AUC) analysis of glucose levels revealed that the STZ-induced diabetic control group had an AUC of 81,695.50 ± 9059.99 mg/dL × min, whereas the groups treated with BCS at 50, 100, and 200 mg/kg showed significantly lower AUCs of 37,941.60 ± 4284.12 mg/dL × min, 25,319.40 ± 1801.86 mg/dL × min, and 19,607.25 ± 2250.01 mg/dL × min (*p* < 0.01), respectively. Notably, the BCS-treated group at 100 mg/kg demonstrated an effect comparable to that of glibenclamide (5 mg/kg, 26,342.54 ± 3683.99 mg/dL × min) (Figure 5).

The OGTT results also indicate that BCS may be highly effective in the short-term regulation of postprandial blood glucose levels, making it a valuable tool for hyperglycemic inhibition. This supports the therapeutic promise of BCS in controlling blood glucose levels, highlighting the synergistic contributions of TQ and insoluble fiber in enhancing glycemic control and mitigating diabetes-related damage [22]. Additionally, the improvement in glucose homeostasis induced by BCS was confirmed through the OGTT results.

#### 3.2.4. Improvement in Insulin Secretion Following BCS Treatment

**Figure 6:**
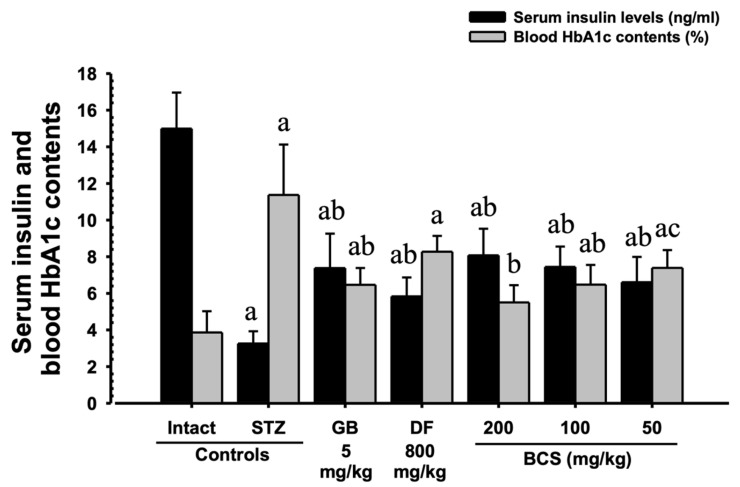
Changes in serum insulin and HbA1c levels. ^a^ *p* < 0.01 as compared with intact vehicle control by DT3 test; ^b^ *p* < 0.01 and ^c^ *p* < 0.05 as compared with STZ control by DT3 test.

After 28 days of BCS administration, serum insulin levels showed a significant increase in the BCS-treated groups compared to the STZ-induced diabetic control group (3.26 ± 0.67 μU/mL). The insulin levels were 6.61 ± 1.38 μU/mL (50 mg/kg), 7.45 ± 1.11 μU/mL (100 mg/kg), and 8.08 ± 1.45 μU/mL (200 mg/kg), with all differences being statistically significant (*p* < 0.01). Notably, the BCS-treated group at 100 mg/kg exhibited an effect comparable to that of glibenclamide (5 mg/kg), which had serum insulin levels of 7.38 ± 1.88 μU/mL. This suggests that BCS enhances pancreatic β-cell function, promoting insulin secretion and thereby contributing to improved glycemic control (Figure 6).

#### 3.2.5. Reduction in HbA1c Levels Following BCS Treatment

In the STZ-induced diabetic model, HbA1c levels were significantly reduced in all BCS-treated groups compared to the STZ-induced diabetic control group (11.37 ± 2.75%). The HbA1c levels were 7.39 ± 0.97% (50 mg/kg), 6.47 ± 1.08% (100 mg/kg), and 5.50 ± 0.94% (200 mg/kg), with all reductions being statistically significant (*p* < 0.01).

#### 3.2.6. Effects of BCS on the Pancreas Caspase-3, TGF-β1, and IL-1β mRNA Expressions

In the pancreas tissue of STZ-induced diabetic rats, the levels of the apoptotic factor caspase-3, the fibrosis marker TGF-β1, and the proinflammatory marker IL-1β were significantly reduced in all BCS-treated groups (50, 100, and 200 mg/kg) compared to the STZ-induced diabetic control group (*p* < 0.05). Additionally, the BCS-treated group at 100 mg/kg showed an effect similar to that of glibenclamide (5 mg/kg) in reducing these markers, further supporting the therapeutic potential of BCS in modulating apoptotic, fibrosis, and inflammatory pathways (Table 2).

#### 3.2.7. Effects of BCS on Pancreatic Histopathology and Insulin-Positive Cell Count

**Figure 7:**
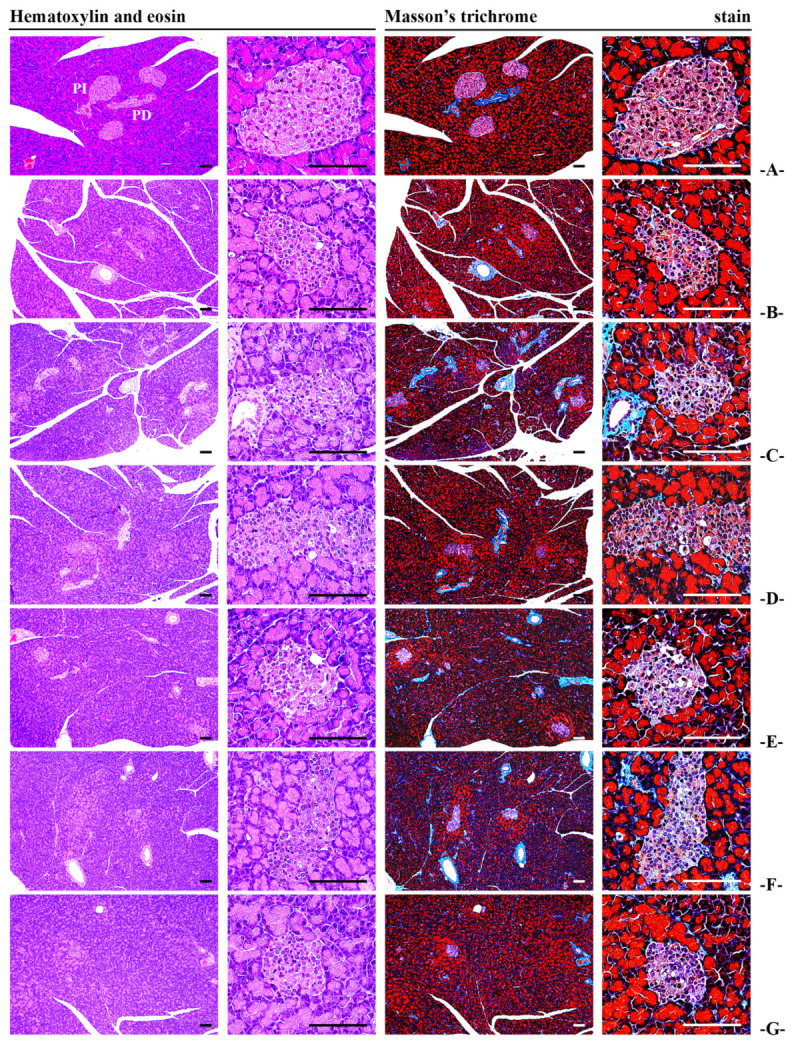
General histological images of the pancreas ((**A**) intact vehicle control rats, (**B**) STZ control rats, (**C**) gilbenclamide 5 mg/kg administered STZ-treated rats, (**D**) dietary fiber 800 mg/kg administered STZ-treated rats, (**E**) BCS 200 mg/kg administered STZ-treated rats, (**F**) BCS 100 mg/kg administered STZ-treated rats, and (**G**) 50 mg/kg administered STZ-treated rats; PI, pancreatic islet; PD, pancreatic duct; scale bars: 80 μm).

**Figure 8:**
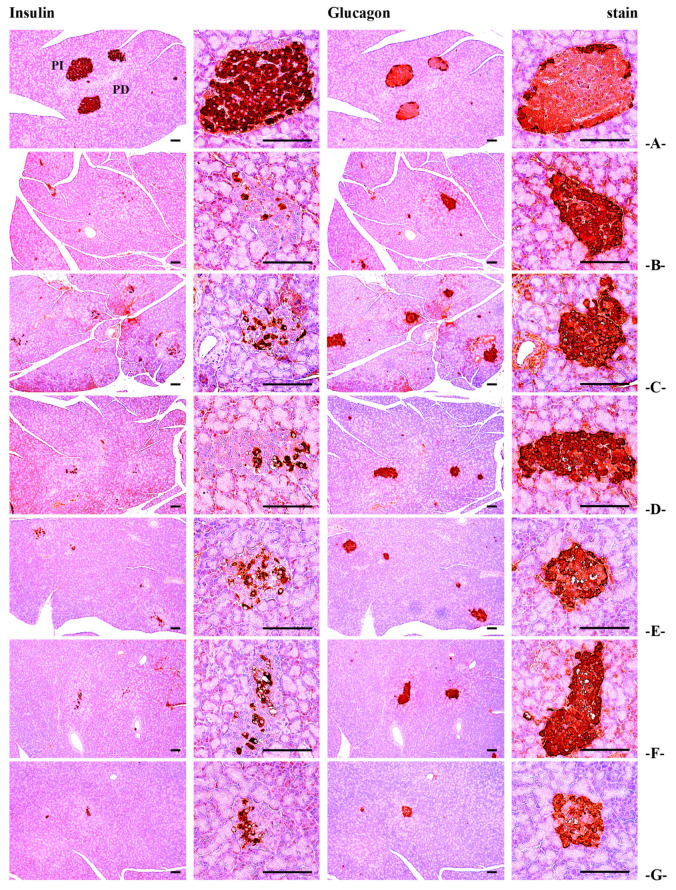
Insulin and glucagon immunoreactive cell images of the pancreas ((**A**) intact vehicle control rats, (**B**) STZ control rats, (**C**) gilbenclamide 5 mg/kg administered STZ-treated rats, (**D**) dietary fiber 800 mg/kg administered STZ-treated rats, (**E**) BCS 200 mg/kg administered STZ-treated rats, (**F**) BCS 100 mg/kg administered STZ-treated rats, and (**G**) 50 mg/kg administered STZ-treated rats; PI, pancreatic islet; PD, pancreatic duct; scale bars: 80 μm).

The number of insulin-positive cells and the insulin/glucagon cell ratio, which were decreased in STZ-induced diabetic rats, were significantly increased in all BCS-treated groups (50, 100, and 200 mg/kg) compared to the STZ-induced diabetic control group (*p* < 0.05). However, there were no significant differences in the number of glucagon-positive cells between the groups (Table 3) (Figure 7 and Figure 8).

### 3.3. Oxidative Stress Mitigation in Liver, Kidney, and Pancreas Following BCS Treatment

In the liver, kidney, and pancreas of STZ-induced diabetic rats, lipid peroxidation (measured by MDA levels) was increased, and antioxidant defense factors (GSH, CAT, and SOD) were decreased. However, these parameters were significantly improved in all BCS-treated groups (50, 100, and 200 mg/kg) compared to the STZ-induced diabetic control group (*p* < 0.05) (Table 4).

### 3.4. Hepatoprotective Effects of BCS: Impact on Liver Weight

#### 3.4.1. The Increase in Both Absolute and Relative Liver Weight

The increase in both absolute and relative liver weight induced by STZ-induced diabetes was significantly reduced in a dose-dependent manner in the BCS-treated groups (50, 100, and 200 mg/kg) (*p* < 0.01) (Table 5).

#### 3.4.2. Effects of BCS on Serum Liver Enzymes and Liver Histopathology

**Figure 9:**
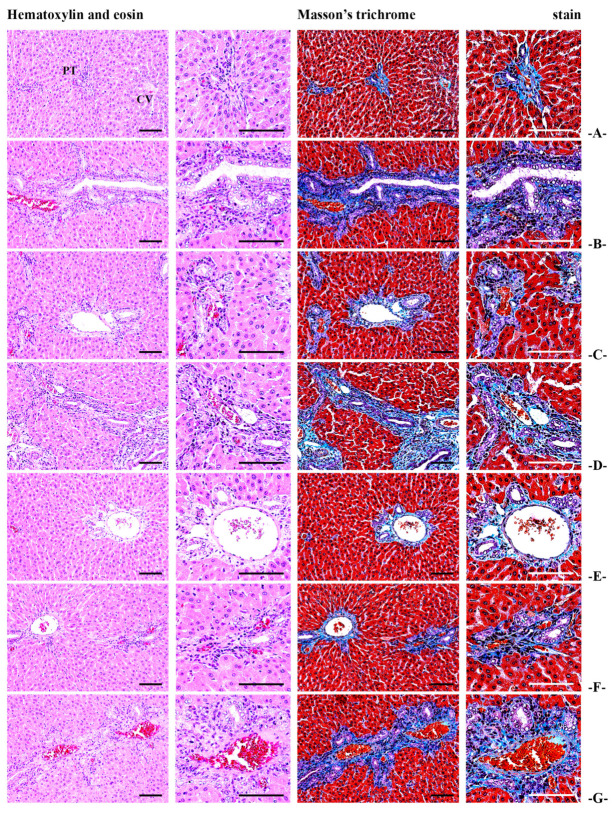
General histological images of the liver ((**A**) in intact vehicle control rats, (**B**) STZ control rats, (**C**) gilbenclamide 5 mg/kg administered STZ-treated rats, (**D**) dietary fiber 800 mg/kg administered STZ-treated rats, (**E**) BCS 200 mg/kg administered STZ-treated rats, (**F**) BCS 100 mg/kg administered STZ-treated rats, and (**G**) 50 mg/kg administered STZ-treated rats; PT, portal triad; CV, central vein; scale bars: 80 μm).

The serum levels of AST, ALT, ALP, LDH, and GGT, which were elevated due to STZ-induced diabetes, showed a significant dose-dependent reduction in the BCS-treated groups (50, 100, and 200 mg/kg) (*p* < 0.01) (Table 10). Similarly, key histopathological abnormalities observed in the livers of STZ-induced diabetic rats, including increased hepatocyte diameters, bile duct proliferation in the portal triad regions, inflammatory cell infiltration, and collagen deposition, were significantly mitigated in a dose-dependent manner in the BCS-treated groups (*p* < 0.01) (Table 6, Figure 9). Furthermore, histopathological analysis revealed a marked reduction in fibrosis, necrosis, and inflammation in the liver tissues of the BCS-treated groups compared to the STZ-induced diabetic control group. These findings highlight the hepatoprotective effects of BCS and its potential to alleviate liver dysfunction associated with diabetes.

#### 3.4.3. Effects of BCS on Hepatic Glucose-Regulating Enzyme Activity

The increase in glucokinase and the decrease in glucose-6-phosphatase and PEPCK levels induced by STZ-induced diabetes were significantly improved in the BCS-treated groups (50, 100, and 200 mg/kg) (*p* < 0.01) (Table 7). Another important aspect of this study is the modulation of hepatic glucose-regulating enzymes by BCS.

### 3.5. Nephroprotective Effects

#### 3.5.1. Effects of BCS on Kidney Weight

The increase in both absolute and relative kidney weight induced by STZ-induced diabetes was significantly reduced in a dose-dependent manner in the BCS-treated groups (50, 100, and 200 mg/kg) (*p* < 0.01) (Table 8).

#### 3.5.2. Effects of BCS on Serum BUN, Creatinine Levels, and Kidney Histopathology

The serum levels of BUN and creatinine, which were increased in STZ-induced diabetic rats, were significantly reduced in a dose-dependent manner in the BCS-treated groups (50, 100, and 200 mg/kg) (*p* < 0.01) (Table 10). Additionally, in the kidney cortex regions, the degenerative areas, VA tubules/100 tubules, and VDA glomerulus/100 glomeruli, which were increased due to STZ-induced diabetes, were significantly reduced in a dose-dependent manner in the BCS-treated groups (50, 100, and 200 mg/kg) (*p* < 0.01) (Table 9) (Figure 8). However, no significant changes were observed in the collagen-occupied regions between the groups.

### 3.6. Hypolipidemic Effects of BCS on Serum Lipid Profile

Serum levels of total cholesterol (TC) and low-density lipoprotein (LDL), which were increased in STZ-induced diabetic rats, and high-density lipoprotein (HDL), which was decreased, showed significant improvement in the BCS-treated groups (50, 100, and 200 mg/kg) (*p* < 0.01). However, no significant improvement was observed in the serum triglycerides (TG) levels, which were elevated due to STZ-induced diabetes (Table 10).

## 4. Discussion

This study underscores the significant therapeutic potential of black cumin seed extract (BCS) as a natural approach for managing diabetes and its associated complications. The anti-hyperglycemic effects of BCS observed in this study were particularly noteworthy, demonstrating efficacy comparable to glibenclamide, a well-established insulin secretagogue. These results highlight the promise of BCS in controlling blood glucose levels, while also shedding light on the synergistic contributions of thymoquinone (TQ) and insoluble fiber in enhancing glycemic control and mitigating diabetes-related damage [22]. The combination of these bioactive components provides a novel and effective strategy in diabetes care, particularly in addressing the limitations of current pharmacological treatments [23].

One of the most striking findings of this study is the preservation of pancreatic islet architecture and the increased number of insulin-positive cells in BCS-treated groups. This indicates that BCS exerts a protective effect on pancreatic β-cell function, which is essential for insulin secretion and glucose regulation. These findings align with previous studies showing that TQ stimulates insulin secretion and protects β-cells from oxidative-stress-induced damage, a common pathway in the progression of diabetes [24,25]. The ability of BCS to preserve the structure and function of pancreatic β-cells is critical, as it may prevent the decline in insulin production in diabetic individuals [26]. Furthermore, reductions in fasting blood glucose and HbA1c levels in BCS-treated rats confirm its capacity to achieve long-term glycemic control, a crucial therapeutic goal for managing diabetes and preventing complications [27].

The STZ-induced diabetes mellitus model is characterized by pancreatic β-cell damage accompanied by a systemic pro-inflammatory state, marked by broad infiltration of macrophages [28]. In the present study, the STZ-induced diabetes model also demonstrated pancreatic β-cell damage resulting from changes in pancreatic islets. The mRNA expression of IL-1β, a key inflammatory mediator in pancreatic β-cells, was significantly increased compared to the vehicle control. This suggests that pancreatic β-cell damage may be attributed to the activation of nuclear factor-κB (NF-κB), leading to the stimulation of inducible nitric oxide synthase (iNOS) and the subsequent generation of nitric oxide (NO), which in turn contributes to pancreatic islet cell death [29,30,31]. Notably, BCS significantly reduced IL-1β mRNA expression in a dose-dependent manner, which is thought to directly promote increased insulin secretion by restoring pancreatic β-cell integrity. This interpretation is supported by the present data, which indicate that caspase-3 and TGF-β1 levels in pancreatic tissue were significantly reduced in a dose-dependent manner by BCS. Consequently, the insulin/glucagon cell ratio in pancreatic islets was significantly increased, further reinforcing this conclusion. Furthermore, in a previous study using a high-fat-diet mouse model [14], it was confirmed that BCS increased the insulin/glucagon cell ratio in pancreatic islets in a dose-dependent manner. This suggests that BCS is effective in improving insulin secretion by restoring pancreatic β-cell damage caused by diabetes mellitus. However, to attain a clearer understanding of the mechanisms by which BCS restores pancreatic β-cell integrity, further studies are needed to investigate its anti-diabetic actions across multiple organs, including the pancreas, intestinal epithelium, skeletal muscle, and liver. These studies should focus on the inhibition of various inflammatory agents, reduction in oxidative stress, and the suppression of apoptosis.

**Figure 10:**
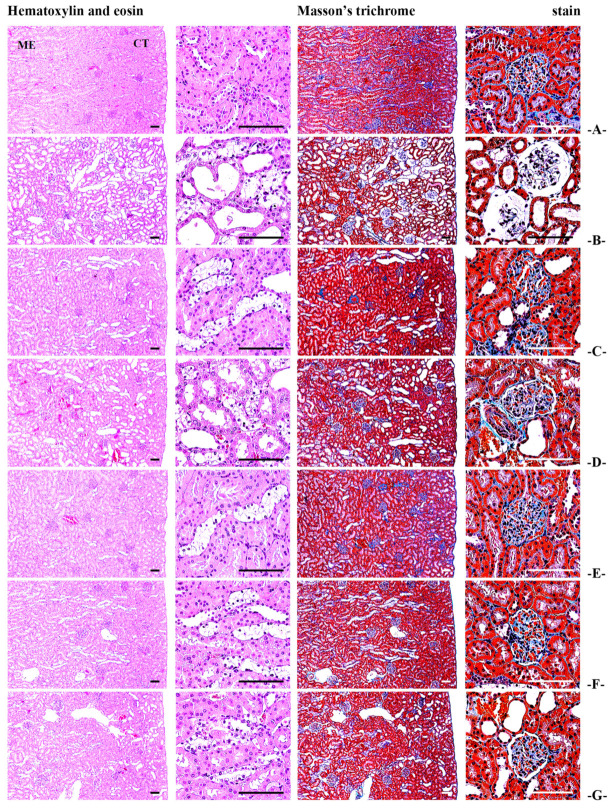
General histological images of the kidney ((**A**) intact vehicle control rats, (**B**) STZ control rats, (**C**) gilbenclamide 5 mg/kg administered STZ-treated rats, (**D**) dietary fiber 800 mg/kg administered STZ-treated rats, (**E**) BCS 200 mg/kg administered STZ-treated rats, (**F**) BCS 100 mg/kg administered STZ-treated rats, and (**G**) 50 mg/kg administered STZ-treated rats; ME, medulla; CT, cortex; scale bars: 80 μm).

Beyond glycemic benefits, BCS exhibited hepatoprotective and nephroprotective effects, as evidenced by histopathological evaluations showing reduced fibrosis, inflammation, and cellular damage in the liver and kidneys of treated rats (Figure 10). These protective effects are likely due to the antioxidant and anti-inflammatory properties of BCS, supported by observed increases in the activities of key antioxidant enzymes such as catalase (CAT) and superoxide dismutase (SOD), along with elevated levels of reduced glutathione (GSH) [32]. Additionally, BCS directly suppressed the expression of genes associated with apoptosis (caspase-3), fibrosis (TGF-β1), and inflammation (IL-1β), further demonstrating its potential to mitigate diabetes-induced tissue damage and promote repair processes [33].

Another important aspect of this study is the modulation of hepatic glucose-regulating enzymes by BCS. Specifically, BCS promoted glycolysis through increased glucokinase (GK) activity and suppressed gluconeogenesis by reducing glucose-6-phosphatase (G6pase) and phosphoenolpyruvate carboxykinase (PEPCK) activities [34]. This dual modulation of glucose metabolism provides a mechanistic explanation for the potent anti-hyperglycemic effects of BCS. By promoting glucose utilization and inhibiting excessive production in the liver, BCS helps restore glucose homeostasis, a key factor in controlling hyperglycemia in diabetic patients [35]. Additionally, the improvement in glucose homeostasis induced by BCS was confirmed through the oral glucose tolerance test (OGTT) results, which suggest its effectiveness in the short-term regulation of postprandial blood glucose levels [22]. 

This hypothesis is supported by the notion that the regeneration of pancreatic β-cells damaged by diabetes mellitus through BCS leads to improved insulin secretion in response to increased blood glucose levels. The secreted insulin then regulates glucose homeostasis by suppressing the mRNA expression of the forkhead transcription factor FOXO1, which controls key enzymes required for gluconeogenesis, such as G6Pase and PEPCK, in the liver [36]. Choi and colleagues [37] reported that increased insulin secretion following food intake can enhance the mRNA expression of small heterodimer partner-interacting leucine zipper protein (SMILE) in the liver, which is necessary for inhibiting gluconeogenesis. This, in turn, suppresses G6Pase and PEPCK, highlighting the importance of efforts to restore impaired liver function in diabetic patients. In our study, liver histomorphometrical analysis and changes in liver weight demonstrated that liver damage caused by diabetes mellitus was alleviated by BCS. These findings suggest that BCS facilitates liver recovery, enabling insulin secreted from the pancreas to bind effectively to insulin receptors on the surface of liver cells. This binding likely contributes to the phosphorylation of the tyrosine residues of downstream mediators such as insulin receptor substrate 1 and 2 (IRS1 and IRS2). Simultaneously, the inhibition of gluconeogenesis-related enzymes G6Pase and PEPCK likely contributed to glucose homeostasis, offering further evidence of BCS’s therapeutic potential.

In addition to its effects on glucose metabolism, BCS demonstrated beneficial effects on lipid metabolism. It reduced levels of low-density lipoprotein (LDL) and total cholesterol while increasing high-density lipoprotein (HDL) levels [38]. These improvements in lipid profiles highlight BCS’s broader metabolic benefits, particularly its potential to address the lipid abnormalities commonly associated with diabetes. By improving lipid profiles, BCS may help reduce the cardiovascular risks often heightened in individuals with diabetes, providing a comprehensive approach to managing the multifaceted complications of the disease [39].

The unique combination of TQ and insoluble fiber in BCS not only enhances glycemic control but also mitigates oxidative stress, reduces inflammation, improves lipid metabolism, and protects organs from damage. These multifaceted effects position BCS as a potential alternative or adjunct to conventional diabetes therapies, offering a holistic approach to disease management [40].

The findings of this study hold significant clinical implications. The ability of BCS to simultaneously address glycemic and non-glycemic complications positions it as a versatile therapeutic agent. Its natural origin and safety profile add to its appeal, especially as an alternative to synthetic drugs that are often associated with adverse effects such as hypoglycemia and gastrointestinal disturbances. The dose-dependent effects observed also suggest that BCS could be tailored to individual patient needs, enhancing its clinical utility. However, the translation of these promising preclinical findings to clinical practice necessitates further research. Detailed investigations into the molecular mechanisms underlying BCS’s therapeutic effects are essential to fully elucidate its mode of action. Clinical trials involving human participants are crucial to validate its safety, efficacy, and long-term benefits. Future studies should also focus on optimizing dosing regimens, exploring potential interactions with existing diabetes medications, and evaluating its applicability across diverse patient populations. Such studies will be critical for establishing BCS as a standard component of diabetes care.

The STZ-induced rat model has been widely used over the past 60 years to study type 1 diabetes mellitus, as it selectively destroys pancreatic islet β-cells [18]. Although this study demonstrated the potential of BCS to restore pancreatic islet β-cells and improve insulin sensitivity in the liver in a model of insulin deficiency and hyperglycemia caused by STZ-induced pancreatic islet β-cell destruction, its findings remain limited. Future studies could explore the generalization of BCS as an anti-diabetic agent by moderating the dose of STZ administration or using a high-fat-diet model that induces approximately 60% β-cell capacity loss, representing type 2 diabetes mellitus. In this study, BCS selectively inhibited the pro-inflammatory marker IL-1β in damaged pancreatic β-cells, but it was insufficient to fully explain the relationship between oxidative stress and apoptosis. Additional research focusing on the interaction between oxidative stress and apoptosis could provide a clearer understanding of the mechanisms behind β-cell proliferation and restoration. The BCS used in this study was a mixture of black cumin seed extract oil and insoluble fiber, which was found to have a relatively higher average TQ content compared to previously reported BCS formulations. However, since no studies have been conducted on its bioavailability during actual ingestion, it is difficult to conclusively determine its superiority over black cumin seed extract oil or black cumin seed extract alone. Therefore, conducting pharmacokinetic and pharmacodynamic studies on TQ following BCS ingestion would provide scientific evidence to objectively evaluate the efficacy of BCS in the treatment of diabetes.

## 5. Conclusions

The present findings suggest that BCS may exhibit anti-hyperglycemic effects in the STZ-induced rat model by promoting the regeneration of damaged pancreatic islet β-cells and enhancing insulin secretion. These effects are likely mediated through the antioxidant and anti-inflammatory properties of BCS, as well as its capacity to address insulin deficiency via pancreatic islet β-cell proliferation. The beneficial impact of BCS was evident in improvements in enzyme activity, protein levels, histological structures, and immunohistochemical markers affected by STZ. Further studies are strongly recommended to assess the long-term efficacy and safety of BCS and to establish its potential as an antidiabetic agent for managing both type 1 and type 2 diabetes mellitus. Future research should focus on elucidating the mechanisms underlying its effects on insulin signaling and insulin resistance.

## Figures and Tables

**Table 1:** Changes in absolute and relative pancreatic weight.

Group	Absolute Pancreas Weight	Relative Pancreas Weight
Controls		
Intact vehicle	1.077 ± 0.104	0.266 ± 0.036
STZ	0.657 ± 0.040 ^a^	0.304 ± 0.017
Reference drug		
GB 5 mg/kg	0.792 ± 0.062 ^ab^	0.304 ± 0.025
DF 800 mg/kg	0.734 ± 0.022 ^a^	0.297 ± 0.014
BCS		
200 mg/kg	0.859 ± 0.120 ^ab^	0.313 ± 0.049 ^b^
100 mg/kg	0.795 ± 0.079 ^ab^	0.307 ± 0.036
50 mg/kg	0.764 ± 0.059 ^ac^	0.300 ± 0.024

Values are expressed as the mean ± SD of 10 rats, g. STZ, streptozotocin; GB, glibenclamide; BCS, black cumin (Nigella sativa L.) seed extract; DF, dietary fiber; THSD; Tukey’s honest significant difference. ^a^ *p* < 0.01 and ^b^ *p* < 0.05 as compared with intact vehicle control by THSD test; ^c^ *p* < 0.01 as compared with STZ control by THSD test.

**Table 2:** Changes in caspase-3, TGF-β1, and IL-1β mRNA expression in pancreatic tissue.

	Item	Pancreas mRNA Expressions		
Group		Caspase-3	TGF-β1	IL-1β
Controls				
Intact vehicle		1.03 ± 0.09	1.03 ± 0.12	1.02 ± 0.11
STZ		2.80 ± 0.33 ^a^	4.98 ± 1.66 ^c^	3.30 ± 0.76 ^c^
Reference drug				
GB 5 mg/kg		1.93 ± 0.33 ^ab^	2.33 ± 0.64 ^ce^	1.78 ± 0.29 ^cd^
DF 800 mg/kg		2.25 ± 0.21 ^ab^	3.19 ± 0.19 ^c^	2.21 ± 0.23 ^ce^
BCS				
200 mg/kg		1.50 ± 0.26 ^ab^	2.16 ± 0.69 ^cd^	1.47 ± 0.25 ^cd^
100 mg/kg		1.92 ± 0.25 ^ab^	2.28 ± 0.57 ^cd^	1.78 ± 0.23 ^cd^
50 mg/kg		2.09 ± 0.20 ^ab^	2.67 ± 0.57 ^ce^	2.00 ± 0.17 ^cd^

Values are expressed as the mean ± SD of 10 rats, relative to control/β-actin mRNA expressions (TGF, transforming growth factor; IL, interleukin). ^a^ *p* < 0.01 as compared with intact vehicle control by THSD test; ^b^ *p* < 0.01 as compared with STZ control by THSD test; ^c^ *p* < 0.01 as compared with intact vehicle control by DT3 test; ^d^ *p* < 0.01 and ^e^ *p* < 0.05 as compared with STZ control by DT3 test.

**Table 3:** Pancreas histomorphometrical analysis.

		Pancreatic Islets					
	Item (Unit)	Numbers/10 mm^2^	Diameters (μm)	Collagen Occupied Regions (%/mm^2^)	Insulin Cells (numbers/mm^2^)	Glucagon Cells (numbers/mm^2^)	Insulin/Glucagon Cell Ratio
Group							
Controls							
Intact vehicle		18.00 ± 3.05	185.62 ± 19.02	1.02 ± 0.11	513.60 ± 96.64	51.00 ± 10.47	10.13 ± 0.68
STZ		2.08 ± 1.14 ^c^	65.80 ± 10.54 ^a^	3.30 ± 0.76 ^c^	13.00 ± 3.68 ^c^	58.20 ± 12.38	0.23 ± 0.06 ^c^
Reference drug							
GB 5 mg/kg		9.80 ± 1.75 ^ce^	105.99 ± 14.77 ^ab^	1.78 ± 0.29 ^ce^	49.00 ± 13.17 ^ce^	56.20 ± 12.25	0.89 ± 0.22 ^ce^
DF 800 mg/kg		6.00 ± 0.94 ^ce^	82.31 ± 10.99 ^a^	2.21 ± 0.23 ^ce^	22.20 ± 1.99 ^ce^	53.70 ± 8.27	0.42 ± 0.06 ^ce^
BCS							
200 mg/kg		13.10 ± 2.28 ^de^	124.11 ± 19.17 ^b^	1.47 ± 0.25 ^de^	61.80 ± 20.54 ^ce^	51.10 ± 12.72	1.25 ± 0.39 ^ce^
100 mg/kg		9.90 ± 1.91 ^ce^	104.87 ± 20.36 ^ab^	1.78 ± 0.23 ^ce^	49.80 ± 11.37 ^ce^	54.20 ± 12.02	0.93 ± 0.15 ^ce^
50 mg/kg		8.20 ± 1.62 ^ce^	93.12 ± 15.65 ^ab^	2.00 ± 0.17 ^ce^	38.00 ± 14.05 ^ce^	54.60 ± 12.08	0.74 ± 0.36 ^cf^

Values are expressed as the mean ± SD of 10 rats. ^a^ *p* < 0.01 as compared with intact vehicle control by THSD test; ^c^ *p* < 0.01 and ^d^ *p* < 0.05 as compared with intact vehicle control by DT3 test; ^b^ *p* < 0.01 as compared with STZ control by THSD test; ^e^ *p* < 0.01 and ^f^ *p* < 0.05 as compared with STZ control by DT3 test.

**Table 4:** Lipid peroxidation and antioxidant defense systems.

	Group	Controls		Reference Drug		BCS		
Item (Unit)		Intact Vehicle	STZ	GB 5 mg/kg	DF 800 mg/kg	200 mg/kg	100 mg/kg	50 mg/kg
Liver								
MDA (nM/g protein)		82.10 ± 16.08	319.00 ± 69.83 ^e^	142.80 ± 28.96 ^eg^	205.30 ± 34.05 ^eg^	120.80 ± 23.01 ^eg^	142.60 ± 30.42 ^eg^	181.80 ± 36.81 ^eg^
GSH (μM/g protein)		166.90 ± 17.74	57.60 ± 22.19 ^a^	121.30 ± 13.73 ^ac^	96.80 ± 13.04 ^ac^	140.70 ± 18.18 ^bc^	123.70 ± 12.02 ^ac^	115.30 ± 13.57 ^ac^
CAT (U/mg protein)		423.80 ± 82.59	168.90 ± 24.92 ^e^	254.40 ± 47.12 ^eg^	215.80 ± 11.37 ^eg^	292.40 ± 44.61 ^fg^	255.40 ± 36.60 ^eg^	234.10 ± 30.78 ^eg^
SOD (U/mg protein)		272.10 ± 49.59	106.00 ± 24.84 ^a^	206.60 ± 30.07 ^ac^	172.10 ± 28.58 ^ac^	230.60 ± 36.43 ^c^	206.80 ± 39.84 ^ac^	181.70 ± 31.52 ^ac^
Kidney								
MDA (nM/g protein)		114.90 ± 29.74	327.10 ± 61.88 ^e^	187.60 ± 30.41 ^eg^	223.30 ± 26.03 ^eg^	161.80 ± 28.94 ^fg^	186.60 ± 28.39 ^eg^	203.90 ± 25.08 ^eg^
GSH (μM/g protein)		155.70 ± 12.65	62.00 ± 10.62 ^a^	105.60 ± 12.37 ^ac^	89.90 ± 12.03 ^ac^	120.50 ± 15.40 ^ac^	104.80 ± 16.04 ^ac^	96.60 ± 12.32 ^ac^
CAT (U/mg protein)		423.60 ± 102.17	192.40 ± 21.54 ^e^	277.90 ± 24.73 ^fg^	250.20 ± 24.54 ^eg^	318.10 ± 55.56 ^g^	276.40 ± 18.73 ^fg^	269.80 ± 23.88 ^fg^
SOD (U/mg protein)		283.60 ± 64.31	117.40 ± 33.63 ^a^	195.00 ± 18.46 ^ac^	165.60 ± 14.86 ^ad^	218.80 ± 32.70 ^c^	196.80 ± 27.94 ^ac^	180.10 ± 18.32 ^ac^
Pancreas								
MDA (nM/g protein)		63.60 ± 15.96	189.40 ± 19.71 ^a^	104.10 ± 18.14 ^ac^	129.50 ± 15.71 ^ac^	84.70 ± 13.98 ^c^	105.50 ± 15.96 ^ac^	115.40 ± 18.61 ^ac^
GSH (μM/g protein)		144.30 ± 25.29	37.40 ± 10.23 ^e^	78.70 ± 14.45 ^eg^	59.80 ± 10.93 ^eg^	97.00 ± 19.07 ^eg^	80.10 ± 15.37 ^eg^	70.90 ± 14.90 ^eg^
CAT (U/mg protein)		396.90 ± 65.32	96.60 ± 31.47 ^a^	206.60 ± 36.77 ^ac^	170.90 ± 21.64 ^ac^	250.70 ± 39.64 ^ac^	207.20 ± 43.72 ^ac^	184.80 ± 27.01 ^ac^
SOD (U/mg protein)		235.70 ± 39.03	46.70 ± 13.57 ^a^	132.70 ± 50.14 ^ac^	98.20 ± 32.38 ^a^	153.90 ± 35.19 ^ac^	132.50 ± 46.23 ^ac^	120.30 ± 44.03 ^ac^

Values are expressed as the mean ± SD of 10 rats (MDA, malondialdehyde; GSH, glutathione; CAT, catalase; SOD, superoxide dismutase). ^a^ *p* < 0.01 and ^b^ *p* < 0.05 as compared with intact vehicle control by THSD test; ^e^ *p* < 0.01 and ^f^ *p* < 0.05 as compared with intact vehicle control by DT3 test; ^c^ *p* < 0.01 and ^d^ *p* < 0.05 as compared with STZ control by THSD test; ^g^ *p* < 0.01 as compared with STZ control by DT3 test.

**Table 5:** Changes in absolute and relative liver weight.

Group	Absolute Liver Weight	Relative Liver Weight
Controls		
Intact vehicle	9.752 ± 0.733	2.403 ± 0.262
STZ	11.564 ± 0.346 ^c^	5.365 ± 0.339 ^a^
Reference drug		
GB 5 mg/kg	9.867 ± 0.684 ^e^	3.790 ± 0.243 ^ab^
DF 800 mg/kg	10.790 ± 0.257 ^de^	4.378 ± 0.268 ^ab^
BCS		
200 mg/kg	9.413 ± 0.646 ^e^	3.425 ± 0.206 ^ab^
100 mg/kg	9.911 ± 0.428 ^e^	3.830 ± 0.270 ^ab^
50 mg/kg	10.094 ± 0.579 ^e^	3.970 ± 0.336 ^ab^

Values are expressed as the mean ± SD of 10 rats, g. ^a^ *p* < 0.01 as compared with intact vehicle control by THSD test; ^b^ *p* < 0.01 as compared with STZ control by THSD test; ^c^ *p* < 0.01 and ^d^ *p* < 0.05 as compared with intact vehicle control by DT3 test; ^e^ *p* < 0.01 as compared with STZ control by DT3 test.

**Table 6:** Liver histomorphometrical analysis.

	Item (Unit)	Hepatocyte Diameters (μm)	Portal Triad Regions		
Group			Bile Ducts (Numbers/mm^2^)	Inflammatory Cells (numbers/mm^2^)	Collagen-Occupied Regions (%/mm^2^)
Controls					
Intact vehicle		14.17 ± 1.00	2.60 ± 1.17	27.00 ± 10.68	1.80 ± 0.83
STZ		25.07 ± 1.17 ^a^	40.00 ± 6.48 ^d^	260.60 ± 76.36 ^a^	15.15 ± 2.73 ^d^
Reference drug					
GB 5 mg/kg		17.84 ± 1.83 ^ac^	13.00 ± 3.43 ^de^	115.00 ± 28.74 ^ac^	7.38 ± 1.31 ^de^
DF 800 mg/kg		20.97 ± 1.42 ^ac^	24.90 ± 3.60 ^de^	175.00 ± 25.88 ^ac^	9.71 ± 0.96 ^de^
BCS					
200 mg/kg		15.88 ± 2.02 ^ac^	7.90 ± 1.91 ^de^	79.40 ± 14.76 ^bc^	3.96 ± 1.15 ^de^
100 mg/kg		17.74 ± 1.67 ^ac^	12.40 ± 3.75 ^de^	114.00 ± 33.56 ^ac^	7.30 ± 1.51 ^de^
50 mg/kg		18.23 ± 1.95 ^ac^	21.60 ± 3.86 ^de^	157.20 ± 23.16 ^ac^	8.68 ± 1.53 ^de^

Values are expressed as the mean ± SD of 10 rats. ^a^ *p* < 0.01 and ^b^ *p* < 0.05 as compared with intact vehicle control by THSD test; ^c^ *p* < 0.01 as compared with STZ control by THSD test; ^d^ *p* < 0.01 as compared with intact vehicle control by DT3 test; ^e^ *p* < 0.01 as compared with STZ control by DT3 test.

**Table 7:** Hepatic glucose regulatory enzyme activities.

	Item	Hepatic Enzyme Activities		
Group		Glucokinase	Glucose-6-Phosphatase	PEPCK
Controls				
Intact vehicle		342.90 ± 109.31	441.10 ± 67.38	169.60 ± 45.06
STZ		118.30 ± 25.19 ^c^	845.30 ± 137.76 ^a^	609.90 ± 102.00 ^a^
Reference drug				
GB 5 mg/kg		192.20 ± 29.98 ^de^	579.20 ± 101.52 ^b^	366.20 ± 85.22 ^ab^
DF 800 mg/kg		167.50 ± 14.61 ^de^	671.60 ± 70.61 ^ab^	453.30 ± 43.63 ^ab^
BCS				
200 mg/kg		212.00 ± 35.44 ^e^	539.23 ± 112.51 ^b^	289.90 ± 70.56 ^ab^
100 mg/kg		193.50 ± 23.85 ^de^	568.00 ± 109.31 ^b^	365.00 ± 71.05 ^ab^
50 mg/kg		180.50 ± 17.11 ^de^	638.40 ± 93.96 ^ab^	402.50 ± 67.92 ^ab^

Values are expressed as the mean ± SD of 10 rats. ^a^ *p* < 0.01 as compared with intact vehicle control by THSD test; ^b^ *p* < 0.01 as compared with STZ control by THSD test; ^c^ *p* < 0.01 and ^d^ *p* < 0.05 as compared with intact vehicle control by DT3 test; ^e^ *p* < 0.01 as compared with STZ control by DT3 test.

**Table 8:** Changes in absolute and relative kidney weight.

Group	Absolute Kidney Weight	Relative Kidney Weight
Controls		
Intact vehicle	1.299 ± 0.045	0.319 ± 0.019
STZ	1.654 ± 0.075 ^a^	0.767 ± 0.050 ^a^
Reference drug		
GB 5 mg/kg	1.412 ± 0.054 ^ab^	0.543 ± 0.031 ^ab^
DF 800 mg/kg	1.504 ± 0.049 ^ab^	0.610 ± 0.039 ^ab^
BCS		
200 mg/kg	1.344 ± 0.044 ^b^	0.490 ± 0.026 ^ab^
100 mg/kg	1.413 ± 0.050 ^ab^	0.546 ± 0.030 ^ab^
50 mg/kg	1.467 ± 0.045 ^ab^	0.577 ± 0.035 ^ab^

Values are expressed as the mean ± SD of 10 rats. ^a^ *p* < 0.01 as compared with intact vehicle control by THSD test; ^b^ *p* < 0.01 as compared with STZ control by THSD test.

**Table 9:** Kidney histomorphometrical analysis.

		Cortex Regions		Glomerulus Regions	
	Item (Unit)	Degenerative Regions (%/mm^2^)	VA Tubules/100 Tubules	VDA Glomerulus/ 100 Glomeruli	Collagen-Occupied Regions (%/mm^2^)
Group					
Controls					
Intact vehicle		1.67 ± 1.12	4.40 ± 1.84	1.50 ± 1.27	4.25 ± 2.03
STZ		68.86 ± 11.01 ^a^	63.70 ± 11.22 ^a^	56.90 ± 10.74 ^a^	6.07 ± 4.39
Reference drug					
GB 5 mg/kg		25.77 ± 10.07 ^ac^	26.20 ± 11.55 ^ac^	22.40 ± 10.31 ^ac^	4.08 ± 3.23
DF 800 mg/kg		44.63 ± 11.08 ^ac^	40.10 ± 10.47 ^ac^	38.30 ± 4.24 ^ac^	5.90 ± 3.71
BCS					
200 mg/kg		11.69 ± 6.87 ^bc^	11.80 ± 4.64 ^bc^	12.00 ± 4.19 ^ac^	4.64 ± 2.95
100 mg/kg		25.02 ± 10.13 ^ac^	24.60 ± 10.06 ^ac^	22.00 ± 10.20 ^ac^	5.02 ± 3.67
50 mg/kg		34.69 ± 10.50 ^ac^	30.20 ± 10.28 ^ac^	27.20 ± 10.35 ^ac^	4.63 ± 2.82

Values are expressed as the mean ± SD of 10 rats. ^a^ *p* < 0.01 and ^b^ *p* < 0.05 as compared with intact vehicle control by DT3 test; ^c^ *p* < 0.01 as compared with STZ control by DT3 test.

**Table 10:** Serum biochemical analysis.

	Group	Controls		Reference Drug		BCS		
Item (Unit)		Intact Vehicle	STZ	GB 5 mg/kg	DF 800 mg/kg	200 mg/kg	100 mg/kg	50 mg/kg
AST(IU/L)		79.40 ± 12.33	336.00 ± 51.04 ^d^	222.20 ± 19.98 ^df^	254.00 ± 15.72 ^df^	173.10 ± 32.05 ^df^	221.20 ± 22.87 ^df^	242.40 ± 22.01 ^df^
ALT (IU/L)		32.50 ± 10.21	248.40 ± 49.86 ^d^	150.20 ± 24.30 ^df^	174.90 ± 15.42 ^dg^	104.50 ± 22.87 ^df^	144.40 ± 17.23 ^df^	162.70 ± 16.08 ^df^
ALP (IU/L)		119.50 ± 43.45	801.30 ± 137.09 ^d^	384.20 ± 85.19 ^df^	537.30 ± 65.36 ^df^	283.90 ± 56.12 ^df^	382.50 ± 103.38 ^df^	452.80 ± 105.50 ^df^
LDH (× 10 IU/L)		74.92 ± 13.02	469.95 ± 133.15 ^d^	192.96 ± 31.07 ^df^	273.59 ± 24.46 ^dg^	144.68 ± 28.47 ^df^	192.58 ± 32.45 ^df^	235.14 ± 29.30 ^df^
GGT (IU/L)		2.70 ± 1.06	9.40 ± 1.65 ^a^	5.40 ± 1.35 ^ac^	6.60 ± 0.70 ^ac^	4.70 ± 0.82 ^ac^	5.30 ± 0.82 ^ac^	6.10 ± 0.99 ^ac^
BUN (mg/dL)		45.80 ± 12.48	148.80 ± 14.18 ^a^	84.80 ± 17.00 ^ac^	108.30 ± 16.05 ^ac^	68.60 ± 16.41 ^bc^	85.30 ± 12.38 ^ac^	94.20 ± 12.27 ^ac^
Creatinine (mg/dL)		0.58 ± 0.13	1.72 ± 0.15 ^a^	1.11 ± 0.16 ^ac^	1.32 ± 0.10 ^ac^	0.92 ± 0.13 ^ac^	1.07 ± 0.14 ^ac^	1.17 ± 0.16 ^ac^
TC (mg/dL)		56.00 ± 11.12	117.70 ± 9.17 ^a^	84.60 ± 14.24 ^ac^	93.80 ± 10.98 ^ac^	72.60 ± 12.29 ^bc^	84.30 ± 14.44 ^ac^	88.30 ± 11.45 ^ac^
TG (mg/dL)		75.60 ± 12.08	89.10 ± 23.44	82.10 ± 13.40	84.20 ± 16.72	80.00 ± 18.50	79.70 ± 17.71	83.10 ± 20.16
LDL (mg/dL)		9.40 ± 2.12	21.30 ± 2.95 ^a^	12.70 ± 1.77 ^ac^	15.50 ± 1.27 ^ac^	10.80 ± 1.32 ^c^	12.20 ± 1.32 ^bc^	13.20 ± 1.55 ^ac^
HDL (mg/dL)		48.80 ± 12.41	11.60 ± 1.58 ^d^	23.40 ± 7.14 ^df^	18.50 ± 4.33 ^dg^	31.00 ± 7.85 ^ef^	24.60 ± 6.36^df^	20.90 ± 3.98^df^

Values are expressed as the mean ± SD of 10 rats (AST, aspartate phosphatase; ALT, alanine phosphatase; ALP, alkaline phosphatase; LDH, lactate dehydrogenase; GGT, gamma-glutamyl transferase; BUN, blood urea nitrogen; TC, total cholesterol; TG, triglycerides; LDL, low-density lipoprotein cholesterol; HDL, high-density lipoprotein cholesterol). ^a^ *p* < 0.01 and ^b^ *p* < 0.05 as compared with intact vehicle control by THSD test; ^d^ *p* < 0.01 and ^e^ *p* < 0.05 as compared with intact vehicle control by DT3 test; ^c^ *p* < 0.01 as compared with STZ control by THSD test; ^f^ *p* < 0.01 and ^g^ *p* < 0.05 as compared with STZ control by DT3 test.

## Data Availability

Data are contained within the article or Supplementary Materials.

## Supplementary Materials

The following supporting information can be downloaded at: https://www.mdpi.com/article/10.3390/antiox14020174/s1. Table S1: Experimental designs used in this study. Table S2: Oligonucleotides for RT-PCR used in this study. Figure S1: Body weight in intact or STZ-induced diabetes.

## Abbreviations

The following abbreviations are used in this manuscript:

ALP	Alkaline phosphatase
ALT	Alanine phosphatase
ANOVA	Analysis of variance
AST	Aspartate phosphatase
BCS	Black cumin seed extract
BUN	Blood urea nitrogen
CAT	Catalase
G6Pase	Glucose-6-phosphatase
GAPDH	Glyceraldehyde-3-phosphate dehydrogenase
GGT	Gamma-glutamyl transferase
GK	Glucokinase
GSH	Glutathione
HbA1c	Hemoglobin A1c
HDL	High-density lipoprotein
HPLC	High-performance liquid chromatography
IL-1β	Interleukin-1 beta
LDH	Lactate dehydrogenase
LDL	Low-density lipoprotein
MDA	Malondialdehyde
OGTT	Oral glucose tolerance test
PEPCK	Phosphoenolpyruvate carboxykinase
ROS	Reactive oxygen species
SOD	Superoxide dismutase
SPF	Specific pathogen-free
STZ	Streptozotocin
TC	Total cholesterol
TG	Triglycerides
TGF-β1	Transforming growth factor-beta 1
TQ	Thymoquinone
VAF	Virus antibody free
