# Supplementary material for: Effects of Black Cumin Seed Extract on Pancreatic Islet β-Cell Proliferation and Hypoglycemic Activity in Streptozotocin-Induced Diabetic Rats
Source: Antioxidants (Basel). 2025 Jan 31;14(2):174. doi: 10.3390/antiox14020174 (PMC11852139; doi:10.3390/antiox14020174)
Supplement: Supplementary file 1 [file antioxidants-14-00174-s001.zip › antioxidants-3434749-supplementary.pdf]

Supplement Data

Table S1. Experimental designs used in this study

Group	Inducer	Treatment (Dosage)	Animal No.
Control	Citrate buffer	Vehicle (distilled water), 5 ml/kg, oral administration	R01~R10
Control	STZ	Vehicle (distilled water), 5 ml/kg, oral administration	R11~R20
Reference	STZ	Glibenclamide 5 m/kg, oral administration	R21~R30
Reference	STZ	Dietary fibers 800 m/kg, oral administration	R31~R40
Active	STZ	BCS 200 mg/kg, oral administration	R41~R50
Active	STZ	BCS 100 mg/kg, oral administration	R51~R60
Active	STZ	BCS 50 mg/kg, oral administration	R61~R70

Table S2. Oligonucleotides for RT-PCR used in this study

Target	5′ – 3′	Sequence	NCBI accession No.
Caspase-3	Sense	GAATGTCAGCTCGCAATGGTAC	NM_012922
	Antisense	AGTAGTCGCCTCTGAAGAAACTAG	
TGF-β1	Sense	ACCAAGGAGACGGAATAC	NM_021578
	Antisense	ACAGCAGTTCTTCTCTGT	
IL-1β	Sense	TGCCACCTTTTGACAGTGATG	NM_031512
	Antisense	AAGCTGGATGCTCTCATCAGG	
β-actin	Sense	CACTGTCGAGTCGCGTCC	NM_031144
	Antisense	CGCAGCGATATCGTCATCCA	

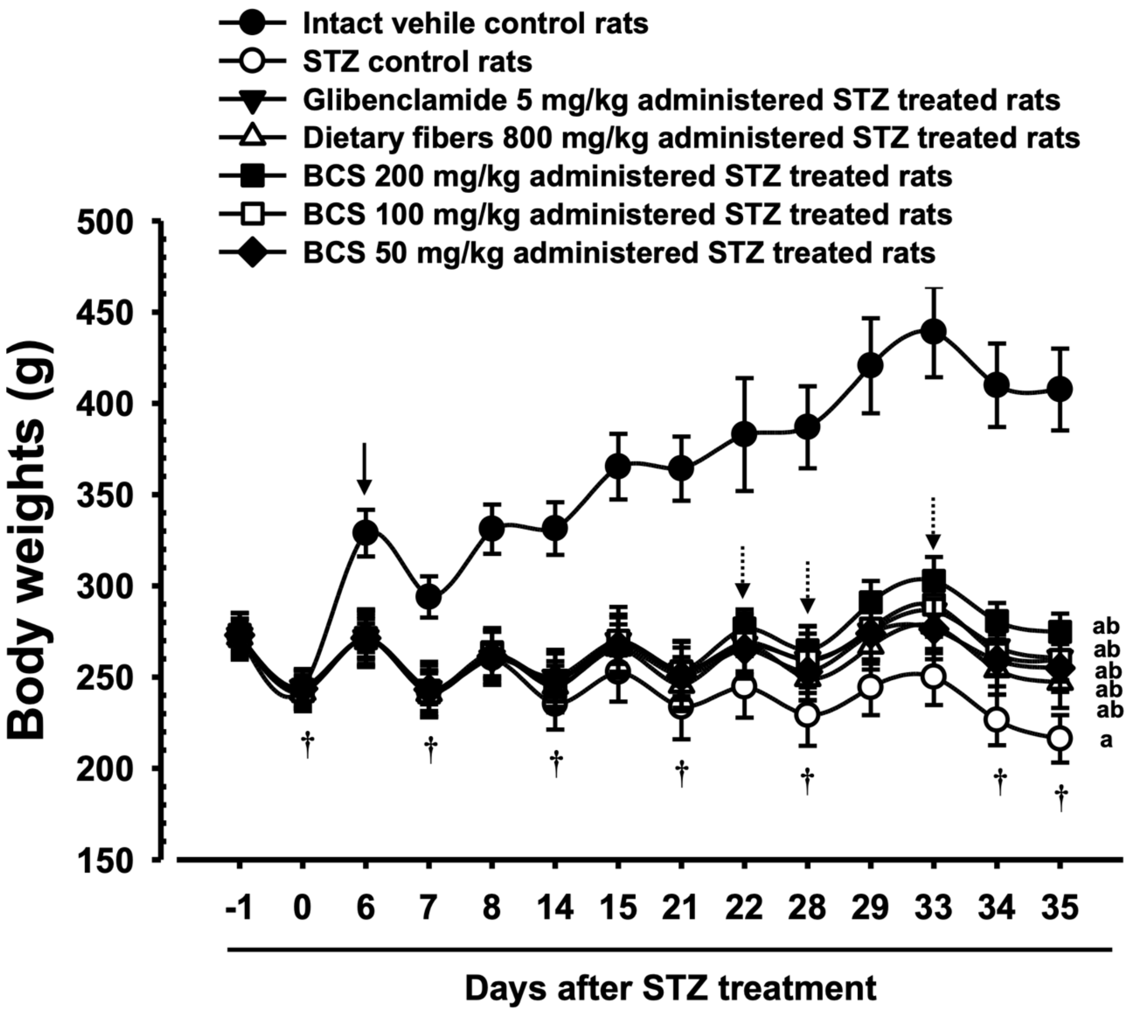

Figure S1. Body weights in intact or STZ-induced diabetes

We selected 10 male Sprague-Dawley rats in each group showing regular body weight changes at 7 days after STZ single intraperitoneally treatment (average 293.90±11.32 g in intact control rats, ranged in 283.00~310.00 g; average 242.37±11.87 g in STZ-induced DM rats, ranged in 216.00~269.00 g, respectively), consequently, STZ control rats showed significant ($p < 0.01$) decreases of body weights from 1 day before initial test article administration as compared with those of intact vehicle control rats (**Arrow**). However, significant ($p < 0.01$) increases of the body weights were detected in glibenclamide 5 mg/kg administered rats from 21 days after initial administration as compared to those of STZ control rats, from 26 days after initial administration in dietary fibers 800 mg/kg treated rats, and from 14, 21 and 21 days after initial administration in BCS 200, 100 and 50 mg/kg administered rats as compared to those of STZ control rats, respectively (**Dot Arrows**).

[a] $p < 0.01$ as compared with intact vehicle control by THSD test

[b] $p < 0.01$ as compared with STZ control by THSD test
